# Case of Small-Pox after Vaccination

**Published:** 1811-09

**Authors:** Arthur Tegart

**Affiliations:** Pall Mall


### Medical and Physical Journal.

VOL. XXVI.] SEPTEMBER, 1811. [NO. 151.

Printed for R. PHILLIPS, by E. Hemsted, Great New Street, Fetter Lane, London.

*To the Editors of the Medical and Physical Journal.*

### *Case of Small-Pox after Vaccination; by* A. TEGART, *Esq.*

GENTLEMEN,

IN November, 1801, I was desired to vaccinate the only son of Sir Henry Martin, bart. of Harley-street, who exhibited all the usual marks of that disease, in the most complete and satisfactory manner; in saying so, at this distance of time, I do not affect to lay a stress upon my particular recollection of the case in question, but I may be permitted to observe, that in a practice tolerably extensive I never allowed any patient to pass through my hands, as having received the benefit of this important discovery, who had not shewn all the signs of the disease, with its usual distinctive characters. A strong and marked eschar now remains on the arm vaccinated, and Sir Henry Martin tells me, that an eminent professional gentleman saw the child during its progress through the disorder, and considered it as a very fine specimen of the complaint. On this subject, therefore, not a doubt rests on my mind, of the patient having had the cow-pox constitutionally at the time above mentioned. From that period until the present time, the child enjoyed good health, except hooping-cough, which he had favourably, and which still continues in some degree. No other infantile disease took place, but at school about a year ago, the tinea capitis was prevalent: he was affected by it, and at this time is not entirely free from its influence.

I should observe here, that both Sir Henry and Lady Martin suffered severely from the small-pox, the former by inoculation, the latter in the natural way, and that they lost their eldest son in that disorder.

On Monday, the 24th of last June, I was desired to see Master Martin, now in his tenth year, and the account I received from Lady Martin was as follows: that on the preceding Saturday he complained of not being well, was drowsy and heavy, but this did not prevent him accompanying his mother to see the apartments at Carlton House on that day. On the following day, Sunday, he walked out as usual, but on his return felt chilly, and declined his dinner; in the evening became hot and restless, and in the night was very feverish, but appeared much better again on Monday morning. In the afternoon of this day, when I first saw him, he complained very much of headach and pains in his limbs; he had just been sick at his stomach, his skin was hot, his eyes flushed, and upon inquiring, I found that his throat was slightly inflamed.

Under these circumstances I desired he might be put to bed, and directed him to take two grains of calomel, and the same quantity of antimonial powder, and that at intervals he might take saline medicines.

On Tuesday, the 25th, I found he had passed a very restless night, with fever and slight delirium; these symptoms all abated during the day, but in the evening increased considerably; he rambled much in the night, and in the morning of Wednesday, the 26th, an eruption was observed on the upper lip and forehead; he coughed a good deal, and the light became painful to him.

With these symptoms, little doubt was then entertained, that the eruption would prove to be the measles. Saline and antimonial medicines were given at this time; in the evening there was a considerable increase of fever, he passed another very restless night with a recurrence of the delirium. In the morning of Thursday, the 27th, he appeared very much better, the fever had greatly abated, but the eruption which now nearly covered the face, had extended to the extremities, and had put on a more distinct form, and conveyed the idea of an aggravated kind of chicken-pock, the pustules appearing to be surrounded by a reddish margin.

On the evening of this day, from an accidental circumstance, I did not see the patient; I was informed, however, that he passed a very restless night.

On Friday, the 28th of June, the 7th day of the attack, and third of the eruption, very little fever, but the pustules increased in size, and were filled with a whitish fluid, and put on so equivocal an appearance, that I began (reluctantly enough, I admit) to consider the disease as the small-pox. A few pustules were discovered on the tongue, upwards of a hundred were on the face, and about twice that number on the the extremities; but it is remarkable, that the trunk of the body was almost entirely free from eruption.

Under these circumstances I mentioned my apprehension to the family, and having early expressed a wish that Dr. Heberden, the family physician, might see the boy, (but whose attendance at Windsor had hitherto prevented it); I again repeated this desire, and in the interim, called on Mr. Moore, the director of the Vaccine Establishment, who had seen Lord Grosvenor's son, under similar circumstances, requesting that he might accompany me to Harley-street the next morning, which he obligingly did.

On Saturday, the 29th, we found the patient much better; the pustules were now well filled, their tops indented, and clearly conveyed to my mind the idea of the most perfect kind of small-pox. From the peculiarities attending the case, however, Mr. Moore at this period entertained some doubts on the subject. In the evening of this day, viz. the 8th day of the attack, and fourth of the eruption, Dr. Heberden saw the case, and hesitated but little in pronouncing the disease to be the small-pox. The lips were swollen, a few pustules on the left eye had inflamed it, but the eyes at any period of the complaint were never closed from swelling of the integuments. The medical treatment, for the last two days, had been merely giving small doses of the sulphate of magnesia in infus. Rosæ once in four or five hours to keep the bowels gently open.

On Sunday, the 30th, the pustules advanced in size, and were becoming yellow; the patient better in all respects. On this day, at the suggestion of Dr. Heberden, and with the consent of Sir Henry and Lady Martin, I inoculated Miss Martin, a child of four years of age, whom I had vaccinated in March, 1808, (and who had been in the house from the commencement of her brother's illness) from some matter taken from him, and the day following I also inoculated a nursery maid in the family who had been previously vaccinated. On Monday, the 1st of July, the sixth day of the eruption, Master Martin had advanced rapidly towards recovery. The eruption on the face, when it commenced, began to turn; he sat up for several hours in the evening. On Tuesday, the 2d of July, the 7th since the eruption, he was convalescent, went into an adjoining room, and remained up the whole day: and on Wednesday, the 3d of July, the 8th day of the eruption, he was found perfectly well, and amusing himself as boys of that age are generally accustomed to do. The pustules were now falling off rapidly. From this time Dr. Heberden and Mr. Moore discontinued their visits,

 and and the boy was sent out of town perfectly well, and with scarcely any diminution of his strength.

On the first, second, and third day after inoculating Miss Martin and the nursery maid, the parts punctured appeared to inflame rapidly, but from that period the local inflammation diminished, and in a few days after entirely disappeared,

I have thus, Gentlemen, (I fear at an inexcusable length) detailed to you the particulars of a case, which I confess, during its progress, occasioned me considerable anxiety, not on account of the patient, whom I at no period of the complaint considered in danger, but as it has served, by the result, to prove (with some few instances which have occurred to others) that certain constitutions retain a susceptibility of receiving the small-pox contagion, after having passed the vaccine disease; and consequently, that it has weakened the faith I had hitherto entertained of the prophylactic power of the cow-pox in securing such constitutions against the small-pox, which, I believe, it must now be admitted, is not the case to the fullest extent of the word. But when it is recollected that these instances are very few, and that they only occur when a strong idiosyncracy or predisposition to small-pox has manifested itself either in the parents or relatives of the patient; and that in almost every case where the small-pox has followed vaccination, it has always been a mild and mitigated disease, and shorter in duration, and, I believe, in scarcely any instance fatal; surely a decided preference must be given to the discovery of the practice of vaccination, which I have always considered, and continue to think, the greatest blessing to the human race, from its peculiar mildness, from its being unattended with fever or eruption, and above all, from its being a disease which is not communicable through the medium of the atmosphere.

I remain, Gentlemen,

Your obedient humble Servant,

ARTHUR TEGART.

*Pall Mall, July* 8, 1811.

*P.* S. It may not be of any importance to know the particular mode of practice of an individual, but I would beg leave to state here, that I have for some time past adopted the idea suggested by Mr. Bryce, of Edinburgh, in communicating the vaccine disease by what he calls the test, viz. on the 3d, 4th, or 5th day, or as soon as a fluid can be found in the punctured part, taking it from thence and inserting it again in the other arm of the patient; if the constitution be affected by the matter first inserted, the phenomenon presenting itself will be, that the part *last* punctured takes on such active inflammation, that on the 10th day it overtakes, and is is as far advanced as the *first*, with the same circular efflorescence (in miniature) surrounding it. This I consider an additional security of the patient having passed through the vaccine disease.

